# Estimating distemper virus dynamics among wolves and grizzly bears using serology and Bayesian state‐space models

**DOI:** 10.1002/ece3.4396

**Published:** 2018-08-05

**Authors:** Paul C. Cross, Frank T. van Manen, Mafalda Viana, Emily S. Almberg, Daniel Bachen, Ellen E. Brandell, Mark A. Haroldson, Peter J. Hudson, Daniel R. Stahler, Douglas W. Smith

**Affiliations:** ^1^ U.S. Geological Survey Northern Rocky Mountain Science Center Bozeman Montana; ^2^ Boyd Orr Centre for Population and Ecosystem Health Institute of Biodiversity, Animal Health and Comparative Medicine College of Medical, Veterinary and Life Sciences University of Glasgow Glasgow UK; ^3^ Montana Fish, Wildlife and Parks Bozeman Montana; ^4^ Montana Natural Heritage Program Helena Montana; ^5^ Department of Biology Huck Institutes of the Life Sciences Pennsylvania State University University Park Pennsylvania; ^6^ Yellowstone Wolf Project Yellowstone National Park, National Park Service Gardiner Wyoming

**Keywords:** cross‐species transmission, hierarchical models, serology, wildlife disease

## Abstract

Many parasites infect multiple hosts, but estimating the transmission across host species remains a key challenge in disease ecology. We investigated the within and across host species dynamics of canine distemper virus (CDV) in grizzly bears (*Ursus arctos*) and wolves (*Canis lupus*) of the Greater Yellowstone Ecosystem (GYE). We hypothesized that grizzly bears may be more likely to be exposed to CDV during outbreaks in the wolf population because grizzly bears often displace wolves while scavenging carcasses. We used serological data collected from 1984 to 2014 in conjunction with Bayesian state‐space models to infer the temporal dynamics of CDV. These models accounted for the unknown timing of pathogen exposure, and we assessed how different testing thresholds and the potential for testing errors affected our conclusions. We identified three main CDV outbreaks (1999, 2005, and 2008) in wolves, which were more obvious when we used higher diagnostic thresholds to qualify as seropositive. There was some evidence for increased exposure rates in grizzly bears in 2005, but the magnitude of the wolf effect on bear exposures was poorly estimated and depended upon our prior distributions. Grizzly bears were exposed to CDV prior to wolf reintroduction and during time periods outside of known wolf outbreaks, thus wolves are only one of several potential routes for grizzly bear exposures. Our modeling approach accounts for several of the shortcomings of serological data and is applicable to many wildlife disease systems, but is most informative when testing intervals are short. CDV circulates in a wide range of carnivore species, but it remains unclear whether the disease persists locally within the GYE carnivore community or is periodically reintroduced from distant regions with larger host populations.

## 1 INTRODUCTION

Estimating disease transmission across host species remains a key challenge in disease ecology and has important implications for identifying host species or populations that act as reservoirs and optimal control efforts (Haydon, Cleaveland, Taylor, & Laurenson, 2002; Viana et al., 2014). Canine distemper virus (CDV) is one of the most important pathogens of wild carnivores and domestic dogs worldwide (Deem, Spelman, Yates, & Montali, 2000). Canine distemper virus is an example of a disease agent that infects multiple host species, but the role of different host species in sustaining the virus over time is unclear (but see Craft, Hawthorne, Packer, & Dobson, 2008; Viana et al., 2015). More appropriately called carnivore distemper virus, CDV infects a wide range of host species in the *Canidae*,* Ursidae*,* Felidae*,* Mustelidae*,* Procyonidae*,* Hyaenidae*, and *Viverridae* families (Deem et al., 2000). Canine distemper virus is an acute, highly transmissible, and immunizing pathogen similar, in many respects, to other morbilliviruses like measles and rinderpest (Greene & Appel, 2006). Measles requires a relatively large host population (approximately 300,000 or more) to provide the continuous supply of susceptible hosts for the pathogen to persist (Bartlett, 1957, 1960; Bolker & Grenfell, 1995). The higher turnover rate of carnivores compared to humans may allow for persistence of CDV at lower populations, but model estimates suggest that 50,000–100,000 carnivores would be required for even a fifty percent chance of persisting for a period of 10 years (Almberg, Cross, & Smith, 2010). If we consider coyotes as a dominant host, these estimates would still likely translate to an area several times larger than the Greater Yellowstone Ecosystem (GYE). Although the GYE retains an intact community of large and mesocarnivores, they are at relatively low densities for CDV persistence, and most domestic dogs are vaccinated. Thus, CDV likely requires either large areas or intermittent introductions from other regions where mesocarnivores may be more abundant (Almberg et al., 2010).

Three purported CDV outbreaks have occurred in the gray wolf (*Canis lupus*) population of Yellowstone National Park (YNP) since their reintroduction in 1995; all three coincided with significant pup mortality (Almberg, Mech, Smith, Sheldon, & Crabtree, 2009; Almberg et al., 2010; Stahler, Macnulty, Wayne, Vonholdt, & Smith, 2013). During these outbreaks, coyotes (*Canis latrans*) and cougars (*Puma concolor*) were also exposed to CDV (Almberg et al., 2009); however, comparable information is lacking for bear populations of the GYE. Clinical signs of morbidity have been observed in a black bear (*Ursus americanus*) recovered in Pennsylvania (Cottrell, Keel, Brooks, Mead, & Phillips, 2013) and surveys in Alaska showed that both black and grizzly bears (*Ursus arctos*) were exposed to CDV (Chomel, Kasten, Chappuis, Soulier, & Kikuchi, 1998).

**Figure 1:**
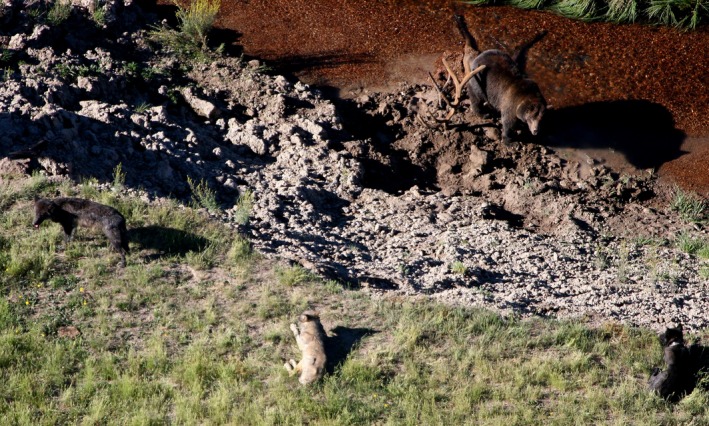
Grizzly bear and wolf interaction at a carcass site is a potential avenue for cross‐species transmission of canine distemper virus.Credit: NPS Photo/D. Stahler [Colour figure can be viewed at http://wileyonlinelibrary.com]

Canine distemper virus is transmitted by close contact via aerosols, oral, respiratory, or ocular fluids, but morbilliviruses do not survive long outside the host. Thus, both direct and environmental transmission across carnivore species may be rare because they may not interact frequently. However, feeding on carcasses is a potential avenue for grizzly bears to acquire infections from other taxa, particularly when grizzly bears push wolves off of recent kills (Figure 1). In this study, we assess the correlation in CDV dynamics in wolves and grizzly bears from the GYE using serological data collected over the last 30 years. We hypothesized that grizzly bears would have greater seroprevalence after wolf reintroduction in 1995, and CDV exposure in bears would be correlated to the timing of distemper outbreaks in wolves.

There are several hurdles associated with inferring disease dynamics in wildlife populations. For acute infections, like CDV, it is unlikely that researchers would capture an animal during the short window of time that they are actively infected and shedding the pathogen. Serological assays are an alternative data‐stream that reflect past exposure and not necessarily a current infection, but are not without their own issues. For example, there are likely differences among laboratories; the timing of the infection is known only to an interval (e.g., from birth to the date of the first sample); and optimal threshold values for distinguishing between positive and negatives tests may be unknown (Gilbert et al., 2013). We address some of these issues through the development of a Bayesian state‐space model that allows for diagnostic testing errors, accounts for the uncertainty in the timing of the infection, and estimates the correlation in the latent disease dynamics across two wildlife hosts.

## 2 MATERIALS AND METHODS

### 2.1 Data collection

The National Park Service captured and radio‐collared wolves annually since their reintroduction in 1995, and generally targets breeders and 50% of each year's young, with an emphasis on maintaining contact with each pack. From 1996 to 2014, this resulted in 285 unique wolves (130 females, 155 males) and 319 sera samples (van Manen et al. 2018). The Interagency Grizzly Bear Study Team (IGBST) also captures bears annually across the GYE. From 1984 to 2014, 565 sera samples were obtained from 425 unique grizzly bears (134 females, 291 males). For additional details on the capture and serum neutralization testing for CDV antibody titers see Blanchard (1985) and Almberg et al. (2009). Sera from wolves and bears were tested by the New York State Animal Health Diagnostic Center (Ithaca, NY, USA). To assess the sensitivity of our conclusions to different titer cutoffs, we applied varying serum neutralization (SN) titer thresholds of greater than or equal to 12, 16, or 24 to indicate an exposed individual.

### 2.2 Statistical approach

To estimate annual CDV infection hazards, we used a Bayesian state‐space model to integrate the data streams across the two host species and account for the unknown timing of infection and potential test errors (Heisey et al., 2010; Viana et al., 2015). Our observed data consisted of serological tests from individuals of known age (Supporting Information Figure S1). We assumed no vertical transmission and lifelong antibody titers, a reasonable biological assumptions for morbilliviruses (Greene & Appel, 2006). Let $\Lambda_{r}$ be the cumulative hazard of infection for time period *r* such that $\Lambda_{r}=\int_{r-1}^{r}h\left(u\right)du$ for the instantaneous hazard *h*(*t*), and let $\gamma_{r}=\log\left(\Lambda_{r}\right).$For an individual *i* in species *s* that was born in year *t* and sampled in year *T*, the probability $\rho_{i,s}\left(T_{i},t_{i}\right)$ of exposure can be related to the constant instantaneous hazard as follows:

$$\rho_{i,s}=1-\exp\left(-\sum_{k=t_{i}}^{T_{i}}\Lambda_{s,k}\right)\;\text{or}\;\rho_{i,s}=1-\exp\left(-\sum_{k=t_{i}}^{T_{i}}\exp\left(\gamma_{s,k}\right)\right),$$

where $\gamma_{s,k}$ is the apparent log hazard of infection for time interval *k*. This is closely related to a complementary log–log model used for interval‐censored survival analyses (Heisey et al., 2010; Prentice & Gloeckler, 1978). In our case, we should refer to $\Lambda_{r}$ as an “apparent hazard” because there may be individuals that become exposed and die prior to being sampled. Thus, our exposure estimates are likely biased low. Individuals tested more than once that were negative on the initial encounter would have additional intervals in the dataset for which *t*_*i*_ would be the date of the previous negative test rather than the birth date.

To account for testing errors, let *q* ^+^ represent the probability that the test is positive given previous infection (i.e., sensitivity) and *q* ^−^ represents the probability that the test is negative given no previous infection (i.e., specificity). Accounting for the possibility of testing errors, the probability that individual *i* is observed as seropositive can be expressed as $P\left(x_{i}=1\right)=\rho_{i,s}q^{+}+\left(1-\rho_{i,s}\right)\left(1-q^{-}\right),$and the probability of being observed as seronegative is $P\left(x_{i}=0\right)=\rho_{i,s}\left(1-q^{+}\right)+\left(1-\rho_{i,s}\right)q^{-}$. We then assume that the datum $x_{i,s}$ is drawn from a Bernoulli distribution with success probability $P(x_{i}=1)$.

There are many potential models for how the log hazard, $\gamma_{s,k}$, may vary across species and over time (Table 1). Although we expect monthly infection hazards to vary during an outbreak year, the data were insufficient to estimate that variation. Therefore, we accounted for how individuals entered and left the dataset using a monthly time step, but assumed the monthly infection hazard was constant for the biological year from March through the following February, which aligns with the timing of den emergence for bears and the wolf birth pulse. We explored several different biological models that included or excluded effects of previous years within or across species, as well as the effect of one species on another within a given year (Table 1). As an example, we may model the bear (s=2) log hazard as: $\gamma_{s=2,k}=\beta_{k}+\alpha_{1}\gamma_{s=1,k}$, where $\alpha_{1}$ is the effect of wolves on bears exposure and $\beta_{k}$ is random intercept term for each year. To investigate the directionality of transmission across species, we compared models with wolves affecting bears and vice versa. Our additional models for $\gamma_{s,k}$ are shown in Table 1, and an example of the model code is provided in the Supporting Information.

**Table 1:** Description of the statistical models, prior distribution, and model fit assuming a serum neutralization threshold of ≥16 to estimate canine distemper virus (CDV) dynamics in wolves and grizzly bears

Model #	Description	Infection hazards	Diagnostics	pD	DIC
No diagnostic errors					
3	Wolves affect bears	*γ*_k,s = 2_ = *β*_k,s = 2 _+ *α*_1_ *γ*_k,s = 1_; *γ*_k,s = 1_~ N(−6, 4); *β*_k,s = 2_ ~ N(−6, 4); *α*_1_ ~ N(0,4)	NA	29.1	764.2
2	Species are independent	*γ*_k,s_ ~ N(−6, 4)	NA	28.3	764.8
1	No species effect	*γ*_k_ ~ N(−6, 4)	NA	17.6	928.0
With diagnostic errors					
5.1	Wolves affect bears with diagnostic errors	*γ*_k,s = 2_ = *β*_k,s = 2 _+ *α*_1_ *γ*_k,s = 1_; *γ*_k,s = 1_~ N(−6, 4); *β*_k,s = 2_ ~ N(−6, 4); *α*_1_ ~ N(0,4)	*q* ^+^ ~ Beta(25, 0.5); *q* ^−^ ~ Beta(25,0.5)	18.7	742.4
5.2	Wolves affect bears, alt. priors	*γ*_k,s = 2_ = *β*_k,s = 2 _+ *α*_1_ *γ*_k,s = 1_; *γ*_k,s = 1_~ N(−6, 10); *β*_k,s = 2_ ~ N(−6, 10); *α*_1_ ~ N(0,10)	*q* ^+^ ~ Beta(25, 0.5); *q* ^−^ ~ Beta(25,0.5)	17.5	741.0
5.3	Wolves affect bears, alt. priors	*γ*_k,s = 2_ = *β*_k,s = 2 _+ *α*_1_ *γ*_k,s = 1_; *γ*_k,s = 1_~ N(−6, 10); *β*_k,s = 2_ ~ N(−6, 10); *α*_1_ ~ N(0,10)	*q* ^+^ ~ Beta(10, 0.5); *q* ^−^ ~ Beta(10,0.5)	19.0	743.3
8	Wolves affect bears, time lags	*γ*_k,s = 2_ = *γ*_k‐1,s = 2_ + *γ*_k‐2, s = 2 _+ *α*_1_ *γ*_k,s = 1_; *γ*_k = 1 or 2, s = 1_~ N(−6, 4); *α*_1_ ~ N(0,4)	*q* ^+^ ~ Beta(25, 0.5); *q* ^−^ ~ Beta(25,0.5)	20.8	747.5
7	Bears affect wolves, time lags	*γ*_k,s = 1_ = *γ*_k‐1,s = 1_ + *γ*_k‐2,s = 1 _+ *α*_2_ *γ*_k,s = 2_; *γ*_k = 1 or 2,s = 2_ ~ N(‐6, 4); *α*_2_ ~ N(0,4)	*q* ^+^ ~ Beta(25, 0.5); *q* ^−^ ~ Beta(25,0.5)	21.4	747.5
9	Wolves affect bears next year	*γ*_k,s = 2_ = *γ*_k‐1, s = 2_ + *γ*_k‐2, s = 2 _+ *α*_1_ *γ*_k‐1, s = 1_; *γ*_k = 1 or 2, s = 1_~ N(−6, 4); *α*_1_ ~ N(0,4)	*q* ^+^ ~ Beta(25, 0.5); *q* ^−^ ~ Beta(25,0.5)	20.7	747.7
4	Species are independent	*γ*_k,s_ ~ N(−6, 4)	*q* ^+^ ~ Beta(25, 0.5); *q* ^−^ ~ Beta(25,0.5)	22.8	752.0
6	Bears affect wolves	*γ*_k,s = 1_ = *β*_k,s = 1 _+ *α*_2_ *γ*_k,s = 2_; *γ*_k,s = 2_~ N(−6, 4); *β*_k,s = 1_ ~ N(−6, 4); *α*_2_ ~ N(0,4)	*q* ^+^ ~ Beta(25, 0.5); *q* ^−^ ~ Beta(25,0.5)	24.4	754.5
Uniform priors (with and without diagnostic errors)					
3.U	Wolves affect bears	*γ*_k,s = 2_ = *β*_k,s = 2 _+ *α*_1_ *γ*_k,s = 1_; *γ*_k,s = 1_~ U(−20, 2); *β*_k,s = 2_ ~ U(‐20, 2); *α*_1_ ~ U(−4,4)	NA	32.4	763.5
2.U	Species are independent	*γ*_k,s_ ~ U(−20, 2)	NA	33.1	764.2
5.U	Wolves affect bears with diagnostic errors	*γ*_k,s = 2_ = *β*_k,s = 2 _+ *α*_1_ *γ*_k,s = 1_; *γ*_k,s = 1_~ U(−20, 2); *β*_k,s = 2_ ~U(−20, 2); a_1_ ~ U(−4,4)	*q* ^+^ ~ Beta(25, 0.5); *q* ^−^ ~ Beta(25,0.5)	18.6	743.2
1.U	No species effect	*γ*_k_ ~ U(−20, 2)	NA	23.3	935.6

*k* represented the year from 1 to 44, *s* = 1 for wolves and 2 for grizzly bears.

*γ* is the log hazard of exposure to CDV.

DIC and pD are the Deviance Information Criterion and the effective number of parameters (Spiegelhalter et al.^2002^).

We explored several possible prior distributions for the different parameters. Initially, we assumed the log hazard $\gamma_{s,k}$ was drawn from a Uniform(−20,2) distribution, but we also used a Normal(−6,4) and Normal(−6,10) distribution. Recall that the monthly probability of exposure, ρ, equals 1 − exp(−exp (γ)), and the annualized probability of exposure is then 1 − (1 − ρ)^12^. Thus, these prior distributions for γ translate to average annual probabilities of infection of roughly 0.1–0.4, but the distributions are bimodal with peaks at 0 and 1. For test errors, we assumed that $q^{+}$ and $q^{-}$ were either drawn from a *Beta* (25, 0.5) or *Beta* (10, 0.5) distribution. To determine the requirement of explicitly accounting for test errors, we also ran a model with no testing errors, in which case $P\left(x_{i}=1\right)=\rho_{i,s}$. We assumed the random intercept $\beta_{k}$ parameters in the model were distributed as Normal(0,10) or Normal(0,4), which on a log scale is still relatively uninformative. Finally, we assumed prior distributions for the slope parameters $\alpha_{x}$ as ∼Normal(0,10) and ∼Normal(0,4). We ran models using R version 3.3.2, JAGS version 4.2.0, and the R2jags package version 0.5‐7 (Plummer, 2003; R Development Core Team 2016; Su & Yajima, 2015) for 200,000 Markov Chain Monte Carlo (MCMC) iterations with a burn‐in of 5,000 iterations on three chains. We assessed MCMC convergence with the Gelman–Rubin convergence diagnostic (Gelman & Rubin, 1992). In a few cases $\hat{R}$ > 1.1, but visual inspection of the parameter estimates for the different MCMC chains suggested that the differences were biologically minor and limited to poorly performing models. We compared models based upon the Deviance Information Criteria (DIC) for those models that used the same data and prior distributions (Spiegelhalter, Best, Carlin, & van der Linde, 2002).

## 3 RESULTS

**Figure 2:**
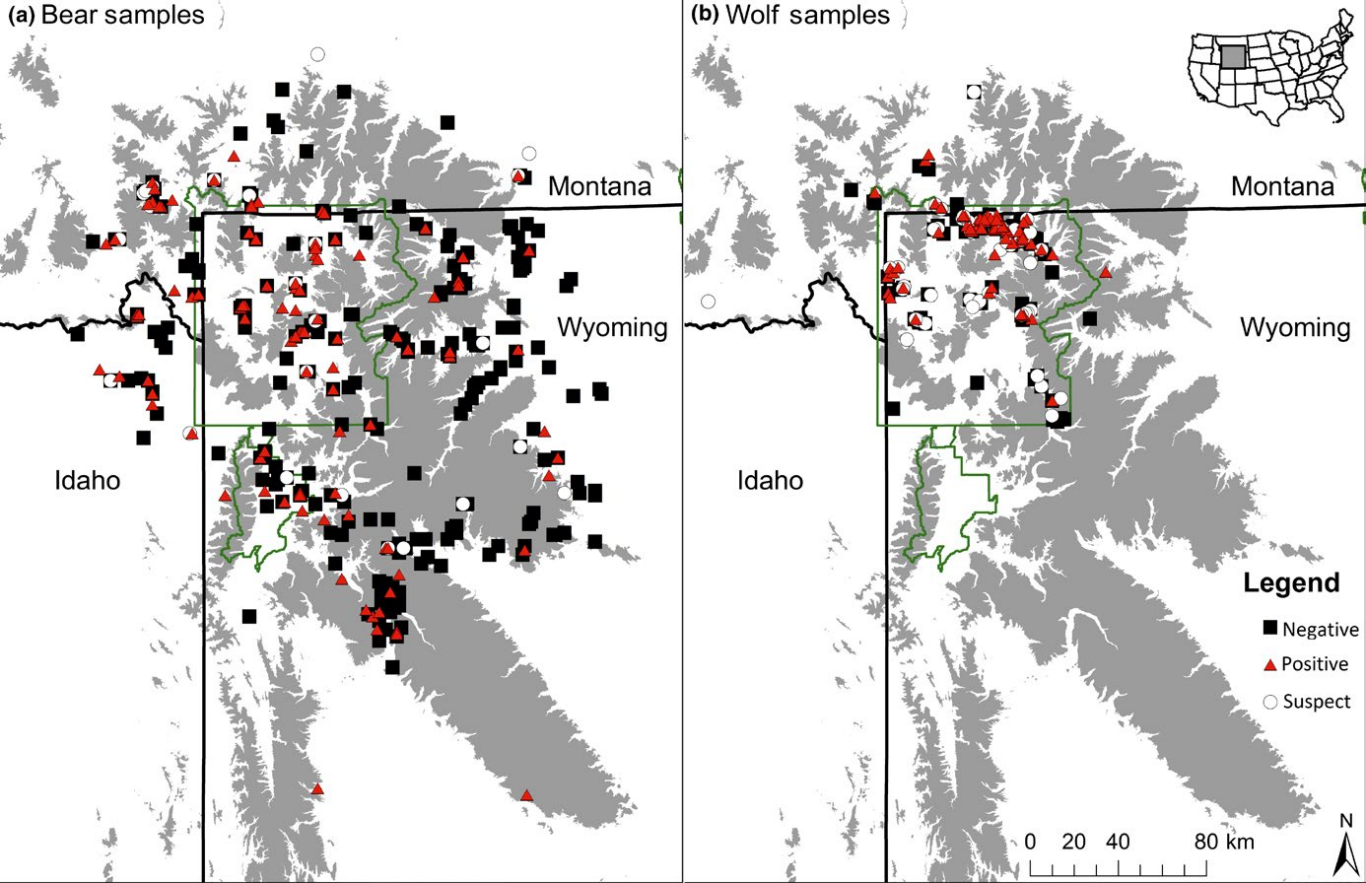
Map of collection locations for grizzly bears (a) and wolves (b) tested for canine distemper virus. Sera neutralization tests that yielded titer values ≥12 and <24 are shown as suspect (white circles). Positive tests (titers ≥24) and negative tests (<12) are shown as red triangles and black squares, respectively. Gray areas are elevations over 2500 m and areas outlined in green indicate, from north to south, Yellowstone National Park, John D. Rockefeller, Jr. Memorial Parkway, and Grand Teton National Park [Colour figure can be viewed at http://wileyonlinelibrary.com]

**Figure 3:**
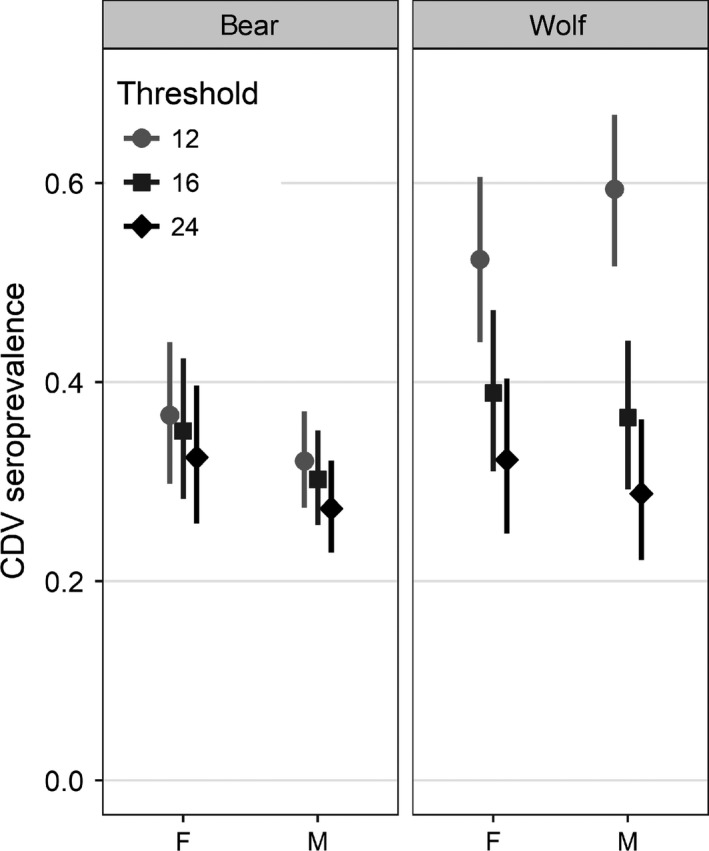
Canine distemper virus (CDV) seroprevalence and 95% binomial confidence intervals for male and female grizzly bears and wolves using different serum neutralization thresholds, Greater Yellowstone Ecosystem, 1984–2014

From 1984 to 2014, we tested 319 wolf and 565 grizzly bear sera samples from across the GYE (Figure 2). About 26% and 6% of the wolf and grizzly bear samples, respectively, yielded SN values between 12 to 24 for CDV (Supporting Information Figure S2). As a result, CDV seroprevalence in wolves varied from around 30% to 60% depending on the titer threshold applied, while grizzly bear seroprevalence was between 30% and 40% (Figure 3).

**Figure 4:**
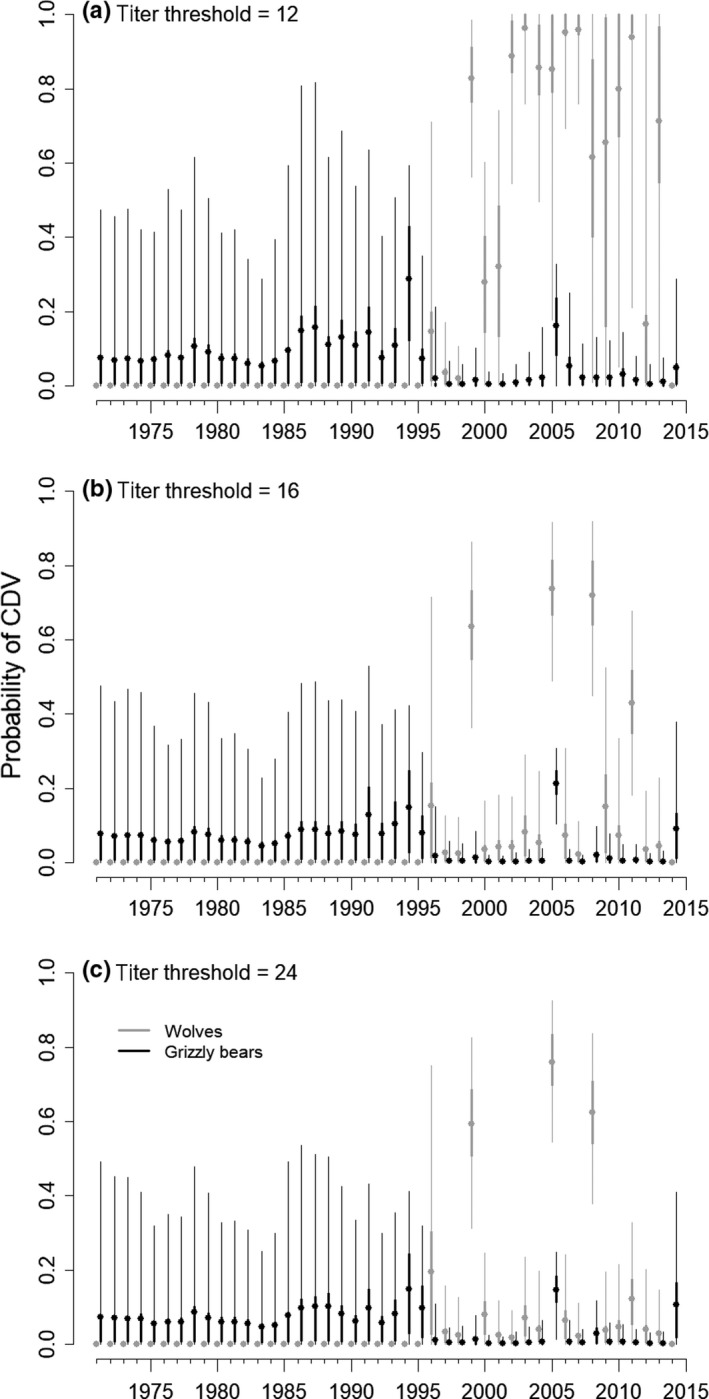
The mean annual canine distemper virus (CDV) exposure probabilities for grizzly bears (black) and wolves (gray) assuming different diagnostic thresholds. Thick and thin lines represent the 50% and 95% credibility intervals, respectively. Estimates were based on Model 5.1 (see Table 1). Wolf estimates were assumed to be zero prior to introduction in 1995

The overall seroprevalence masks the potential variability in the infection hazard over time and does not account for the longer life spans of bears compared with wolves. All models investigated converged well, and based on DIC, models that included an effect of wolf exposure on bear exposure hazards generally performed better (i.e., had lower DIC) than models that assumed independent exposure hazards between the two host species, or that there was an effect of bear exposure on wolf exposure (Table 1). In addition, models also had lower DIC scores when they included the possibility of diagnostic errors (Table 1). Therefore, we focus mostly on models 5.1, 5.2, and 5.3 and present the results of other models in Supporting Information. At titer thresholds of ≥16, our estimates of exposure probabilities highlighted probable CDV outbreaks in wolves in 1999, 2005, 2008, and maybe 2011. These outbreaks of CDV, as indicated by high exposure rates in 1 year followed by several years of low exposure rates, were not apparent in wolves when applying a titer threshold of twelve (Figure 4).

As an acute and highly immunizing disease, one would expect CDV outbreaks to be separated by several years due to herd immunity and a lack of susceptible individuals. Our model estimates based on higher SN thresholds suggested that there were no back‐to‐back outbreak years for wolves (Figure 4). This may result in a negative correlation between exposure rates in 1 year compared to the next. However, when we included one‐ and 2‐year lag effects into the model, those parameters had wide credible intervals with a mode around zero, and these models performed worse than others based on DIC metrics (Table 1). This is probably due to the small number of outbreaks in the time series.

Grizzly bears in the GYE did not appear to have the same large outbreak years as wolves regardless of the titer threshold (Figure 4). Prior to 1996, we had only 16 serological tests on grizzly bears, thus the estimated hazards prior to 1997 largely reflect our prior distributions, and probably does not reflect an actual decline in the CDV hazard following wolf introduction. That said, 38% of those samples collected prior to wolf introduction were seropositive for CDV using a threshold of ≥16. The average duration between birth and testing was 7.7 years for bears compared to 1.7 years for wolves. As a result, the grizzly bear data, in general, were less informative about when they may have been infected and if there were intermittent outbreaks versus more consistent exposures over time.

**Figure 5:**
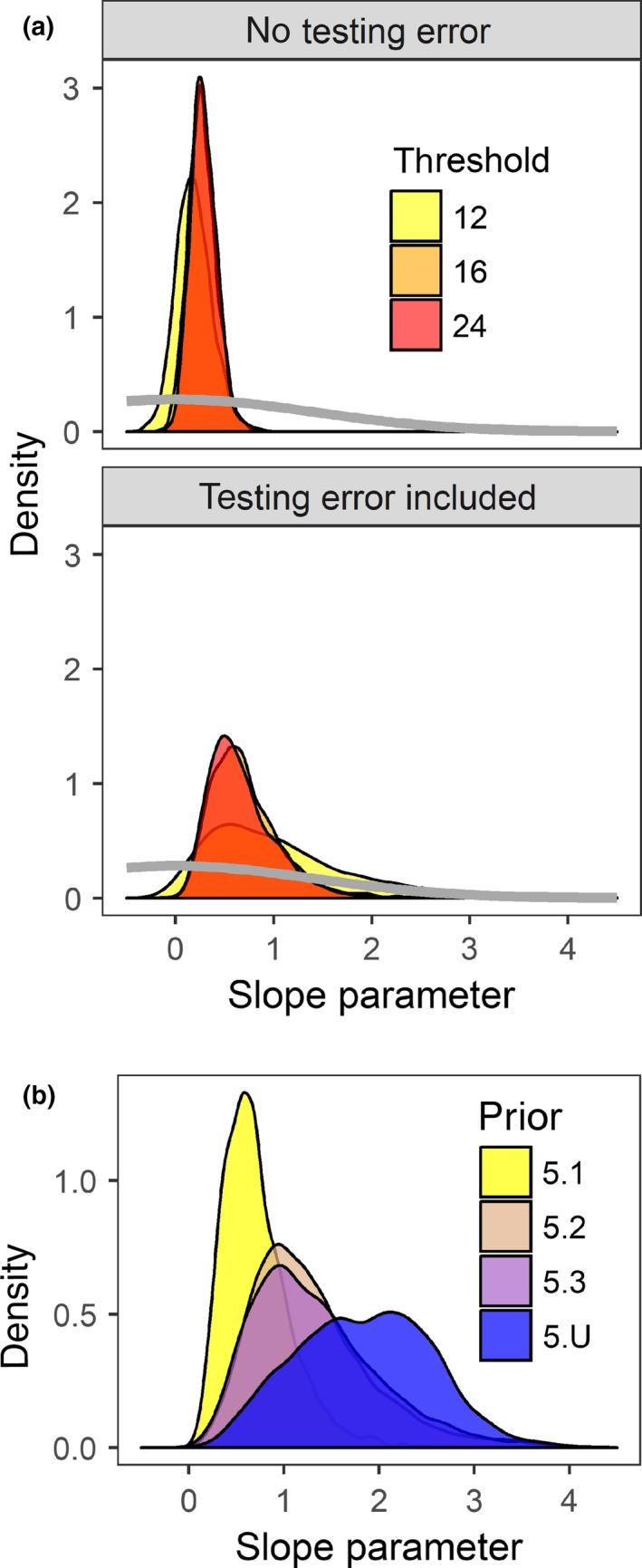
The posterior distributions of the estimated effect of wolf exposures to canine distemper virus on grizzly bear exposure. The slope coefficient ($\alpha_{1}$) depended upon potential diagnostic errors, titer threshold, and the prior distribution. Estimates in (a) were based on Model 5.1, and the gray line is the normal prior distribution with a mean of zero and a variance of four. Assuming a titer threshold of 16 in (b), the posterior distribution of $\alpha_{1}$ increased and became more diffuse in models with less informative priors. See Table 1 for model details [Colour figure can be viewed at http://wileyonlinelibrary.com]

The association between wolves and bears was generally positive, but varied depending on model structure and our prior distributions. There was evidence of a small CDV outbreak in grizzly bears in 2005, coinciding with an outbreak in wolves, but outbreaks in bears were not apparent in either 1999 or 2008 (Figure 4). The estimated slope of the relationship between wolf and bear infections, $\alpha_{1}$, increased as we included more uncertainty into the model, either by including potential testing errors or by using more diffuse prior distributions (Figure 5). Grizzly bears were also exposed to CDV outside of the wolf outbreaks we predicted to have occurred in 1999, 2005, and 2008.

**Figure 6:**
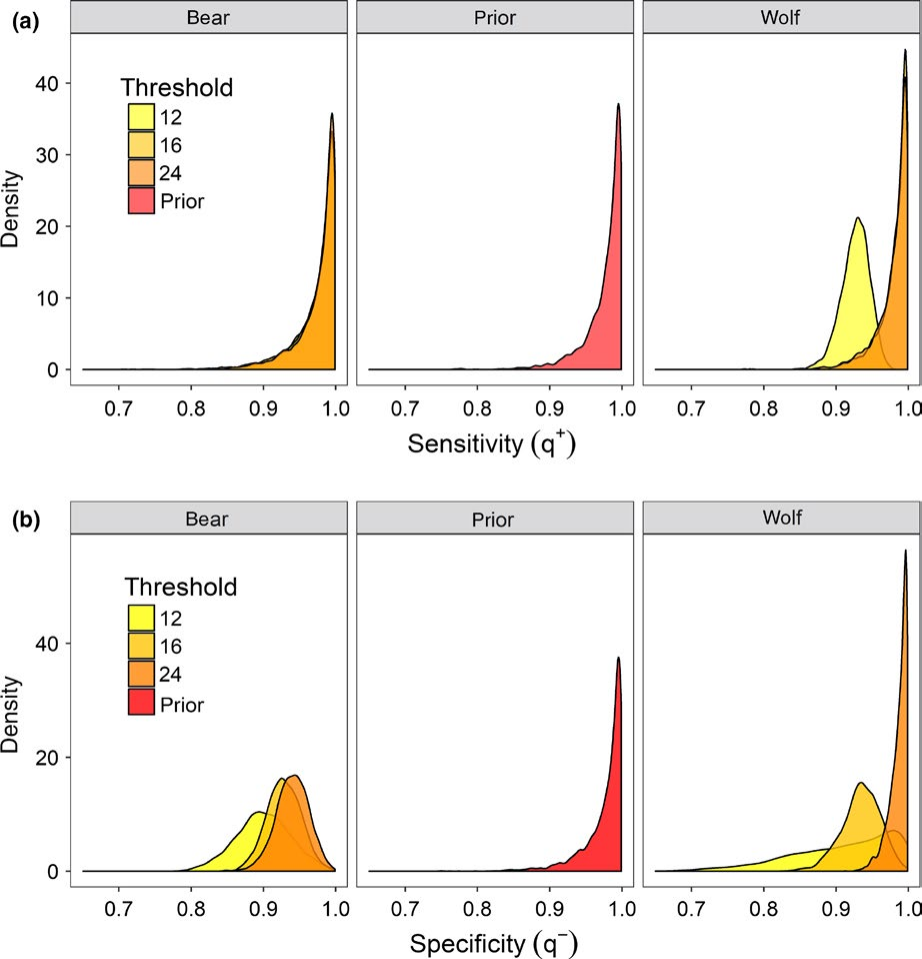
The prior and posterior distributions from Model 5.1 of the sensitivity (*q* ^+^, top row) and specificity (*q* ^−^, bottom row), used to estimate canine distemper virus dynamics in wolves and grizzly bears in the Greater Yellowstone Ecosystem, 1984–2014. The prior distribution for both *q* ^+^ and *q* ^−^ was a Beta(25, 0.5) [Colour figure can be viewed at http://wileyonlinelibrary.com]

The posterior distributions for the sensitivity and specificity parameters $q^{+}$ and $q^{-}$, shifted away from their prior distributions even though there was no direct information included in the analysis about known positive or negative samples (Figure 6). As expected, the estimated specificity, $q^{-}$, declined for both wolves and bears with the titer threshold, as more unexposed individuals would test positive (1‐$q^{-}$). The sensitivity, $q^{+}$, for wolf samples, however, also decreased when the SN threshold was reduced to 12. The posterior distribution on grizzly bear samples, however, did not show similar shifts from the prior distribution (Figure 6).

## 4 DISCUSSION

Our analyses indicated that there were 3–4 CDV outbreaks in wolves in Yellowstone National Park from 1995 to 2014, followed by several years of recovery. This supports the results of Almberg et al. (2010), which identified outbreaks using only samples from juvenile wolves. We expected grizzly bears to be at higher risk of CDV during years when a high proportion of wolves were exposed to CDV because grizzly bears often displace wolves from feeding on carcasses, which is a likely means of transmission between species. Contrary to our predictions, we found that grizzly bears had been exposed to CDV outside of the times of outbreaks in wolves in Yellowstone National Park as well as prior to wolf reintroduction in 1995. These findings are similar to those by Almberg et al. (2010), who found that both cougars and coyotes had evidence of CDV exposure prior to wolf introduction in 1995. We found only weak support for a relationship between the timing of wolf and bear exposures (Figures 4 and 5) as there was evidence of increased CDV exposure in bears during the 2005 wolf outbreak but not otherwise.

Despite relatively long time series and a state‐space hierarchical model, it remained difficult to assess correlations in the disease dynamics among host species, which is an initial step in the direction of estimating the amount of cross‐species transmission. For pathogens with fast growth rates like CDV, an alternative approach to assessing the directionality of transmission may be to assess whether the infections in one species occur prior to the second host species using shorter windows of time (e.g., weekly or monthly). However, this would require a sampling intensity that is unlikely to be achieved in a wildlife host. In our analyses, the magnitude of the wolf effect on bears depended on our prior distributions, suggesting the data were not particularly informative about cross‐species transmission due to more severe interval censoring in the bear disease data (Supporting Information Figure S1) and the limited number of outbreaks in wolves. Interestingly, models that accounted for the possibility of diagnostic test error, estimated stronger connections between the exposure rates of wolves and bears (Figure 5), perhaps by allowing for the possibility that some test‐negative bears may have been positive during wolf outbreaks, and vice versa. At present, the impacts of CDV on grizzly bears are unknown. Clinical disease due to CDV has been reported for black bears (Cottrell et al., 2013), but nothing is known about population‐level impacts. CDV has been related to reducing pup recruitment in wolves from 66% in average years to 18% in outbreak years (Almberg et al., 2009; Stahler et al., 2013). To assess CDV impacts on grizzly bear cub survival, we would need additional data from females with known years of infection (or lack thereof) and their cubs’ survival. Only a few grizzly bears in our data had relatively short testing intervals that could have provided greater precision in estimating the year of infection, but these were often not of females with known reproductive success. A dedicated effort to repeatedly sample female grizzly bears would be required to assess CDV impacts, but would likely take many years to complete.

Our methodological approach addressed key issues associated with serological data and highlighted the importance of accounting for both observational and process error in serological analyses. Several other papers have statistically addressed some of these issues (Buzdugan, Vergne, Grosbois, Delahay, & Drewe, 2017; Conn, Cooch, & Caley, 2010; Heisey et al., 2010; Pepin et al., 2017). Heisey et al. (2010) illustrated how to account for interval censoring, and we built upon this approach to allow for diagnostic testing errors. Conn et al. (2010) accounted for different detection probabilities using a multi‐state mark‐recapture approach, which was further developed by Buzdugan et al. (2017) to allow for multiple diagnostic assays for a single capture event. Finally, Pepin et al. (2017) used data on the dynamics of titer loss within an individual to more precisely estimate the timing of infection of that individual, which also improved population‐level estimates of the force of infection. In our study, we had only a few individuals that tested positive multiple times, and they did not show a strong trend in declining titers that would have allowed for such analysis. Increasing titer thresholds represents an alternative way of incorporating the assumption that recently infected individuals are likely to have higher antibody levels. Our analyses with higher thresholds clearly identified purported outbreaks in wolves that were associated with years of low pup recruitment in 1999, 2005, and 2008 (Almberg et al., 2010; Stahler et al., 2013). This may be because at lower titer thresholds one may get more false positive tests (decreasing specificity) due to nonspecific binding.

Titer thresholds and the inferred sensitivity and specificity of the diagnostic tests interacted in sometimes counterintuitive ways. The estimated test sensitivity for wolves declined at the lowest titer thresholds of 12, even though one would expect the specificity to decline and the sensitivity to increase (Figure 6). We hypothesize that this is just an artifact and not indicative of some biological mechanism, but without data on known infected individuals (perhaps from challenge trials) this is difficult to assess. As expected, however, test specificity declined for both bears and wolves with the lower titer threshold, as more unexposed individuals are observed as test positive. There was no direct information on sensitivity or specificity in this analysis, but the model deviance may decline if an individual's test status can be “re‐assigned” as a potential testing error and allowing the transmission parameter either within or across species to remain high (or low).

Our statistical modeling approach was largely phenomenological in that we did not include mechanistic *S*usceptible‐*I*nfected‐*R*emoved (SIR‐type) disease dynamics (Anderson & May, 1991). This modeling choice was driven by the speed of the disease process relative to the temporal resolution of the data. Most of the CDV disease dynamics for a given outbreak occur within a year, whereas wolves are only captured over the course of a month or two. Bears were captured over a longer time period each year, but the data are still too sparse to investigate weekly or monthly dynamics where a *SIR*‐type model may be more useful. The between‐year CDV dynamics are probably due to changing levels of immunity as well as the timing of introduction events. The introduction events of CDV into the GYE, either from nearby locations or longer‐distances, are unknown.

The carnivore community of the GYE is still probably too small to allow for the local persistence of an acute, highly immunizing pathogen like CDV, particularly in the absence of a large unvaccinated dog population (Almberg et al., 2010; Bartlett, 1957). It is possible that CDV is continuously moving as a wave around the GYE at relatively large spatial scales, such that multiple species are being infected at the same time (Almberg et al., 2010). Most of our wolf data came from the northern portions of Yellowstone NP, whereas the grizzly bear data were collected more broadly across the entire ecosystem (Figure 2). We observed similar dynamics, however, when we limited the data to just the areas north of Yellowstone Lake, and the correlation between bears and wolves did not appear to increase (Supporting Information). Future work on the persistence of CDV should focus on the potential role of mesocarnivores such as skunks (*Spilogale gracilis* and *Mephitis mephitis*) and raccoons (*Procyon lotor*) and acquiring CDV isolates for molecular analyses that may provide information for assessing viral dispersal across large spatial scales.

## CONFLICT OF INTEREST

None declared.

## DATA ACCESSIBILITY

The dataset and code supporting this article have been uploaded to ScienceBase.gov https://doi.org/10.5066/p96e4uck (van Manen et al., 2018).

## ETHICAL STATEMENT

Grizzly bear capture and handling procedures used for this study were reviewed and approved by the respective Animal Care and Use Committees of IGBST partner agencies. Procedures conformed to the Animal Welfare Act and to U.S. Government principles for the use and care of vertebrate animals used in testing, research, and training. Captures were conducted under U.S. Fish and Wildlife Service Endangered Species Permit [Section (i) C and D of the grizzly bear 4(d) rule, 50 CFR17.40 (b)], with additional state research permits for Wyoming, Montana, and Idaho, and National Park Service research permits for Yellowstone and Grand Teton National Parks. Wolves were captured and handled following protocols in accord with applicable guidelines from the American Society of Mammalogists and approved by the National Park Service Institutional Animal Care and Use Committee.

## AUTHOR'S CONTRIBUTIONS

D.W.S., D.R.S., F.T.vM, M.H., E.S.A, the Interagency Grizzly Bear Study Team and the Yellowstone Wolf Project collected the data. P.C.C., M.V., and D.B. conducted the statistical analyses. P.C.C conceived the idea for this article and wrote the initial manuscript. All authors provided critical feedback and contributed to content.
