# Supplementary material for: Estimating distemper virus dynamics among wolves and grizzly bears using serology and Bayesian state‐space models
Source: Ecol Evol. 2018 Aug 5;8(17):8726–35. doi: 10.1002/ece3.4396 (PMC6157674; doi:10.1002/ece3.4396)
Supplement: Supplementary file 1 [file ECE3-8-8726-s001.docx]

Supplementary material:


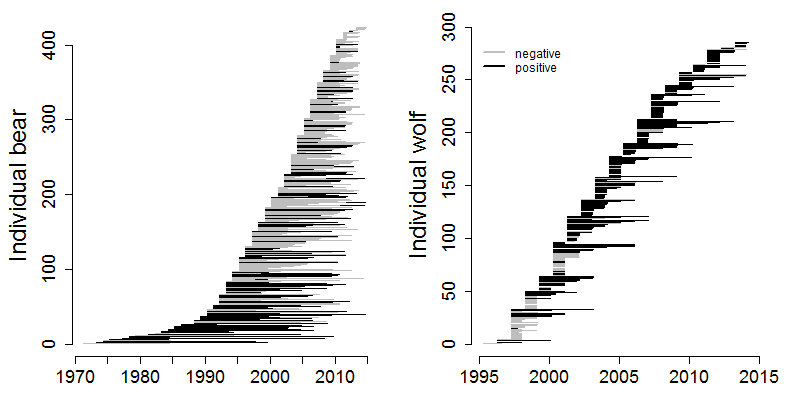

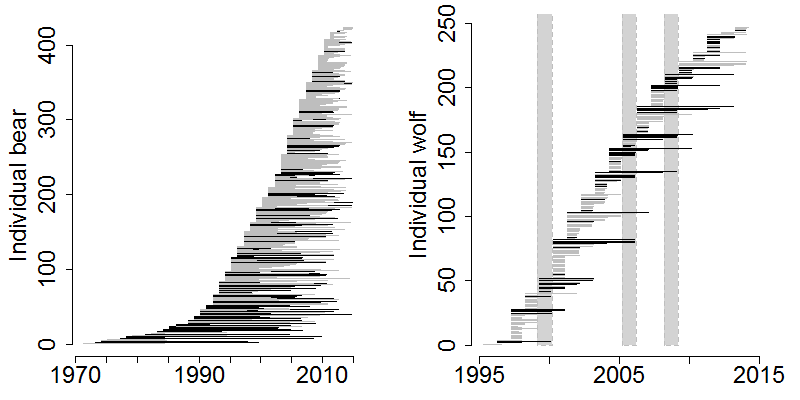


**Figure S1.** Canine distemper virus testing interval data for grizzly bears (left column) and wolves (right column) assuming a titer threshold 12 (top row) or 16 (bottom row), Greater Yellowstone Ecosystem, based on serological data collected during 1984-2014. Each line extends from birth to the last test result. Individuals tested more than once may change from negative (grey lines) to positive, but the timing of the infection is only known to the interval between tests. The grey polygons represent the timing of wolf outbreaks identified by pup mortalities (Almberg et al. 2009).


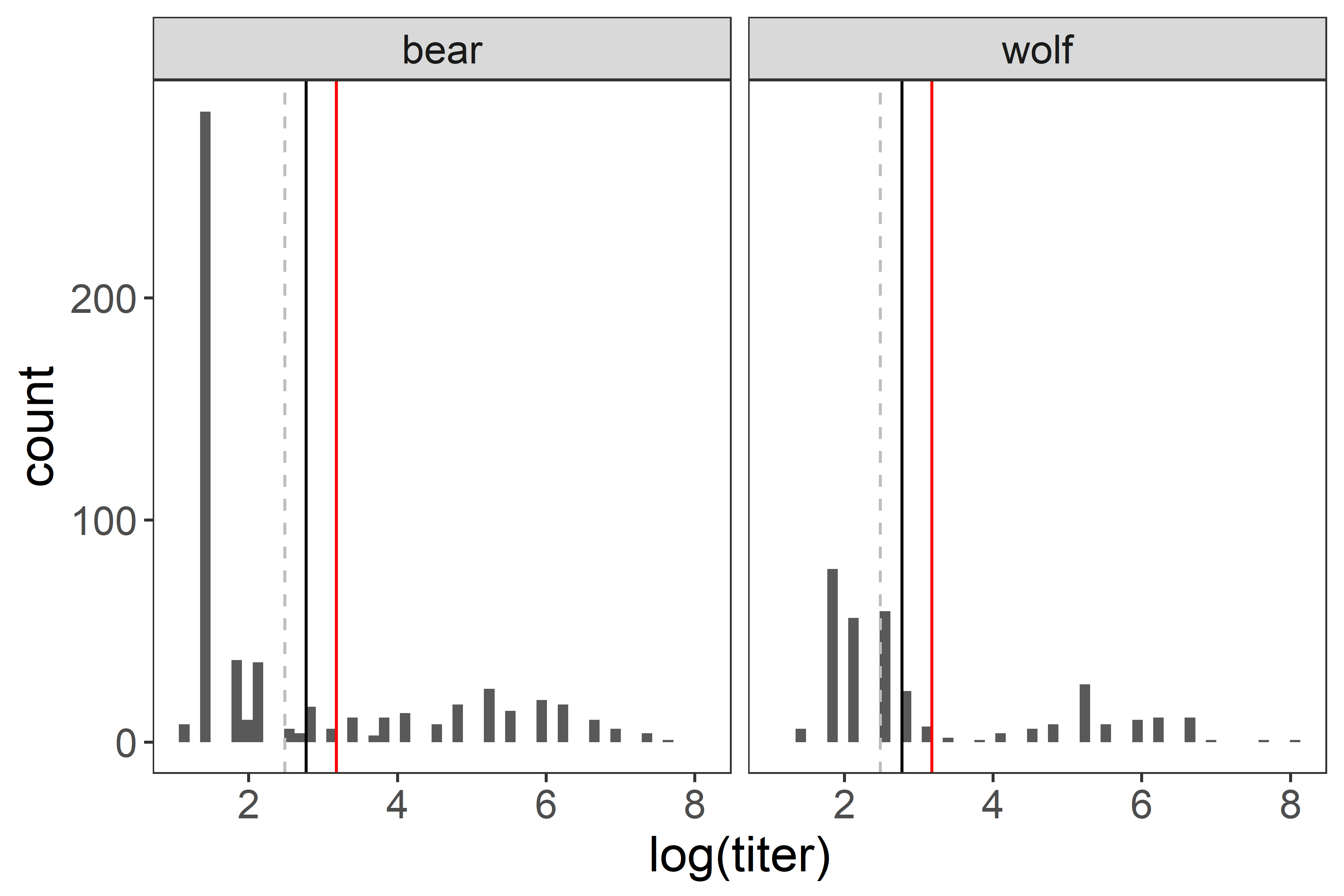


**Figure S2.** Distribution of canine distemper virus antibody titer levels in grizzly bears and wolves, Greater Yellowstone Ecosystem, 1984-2014. Serum neutralization titer thresholds of 12, 16 and 24 are included as dashed, black and red lines, respectively.

##


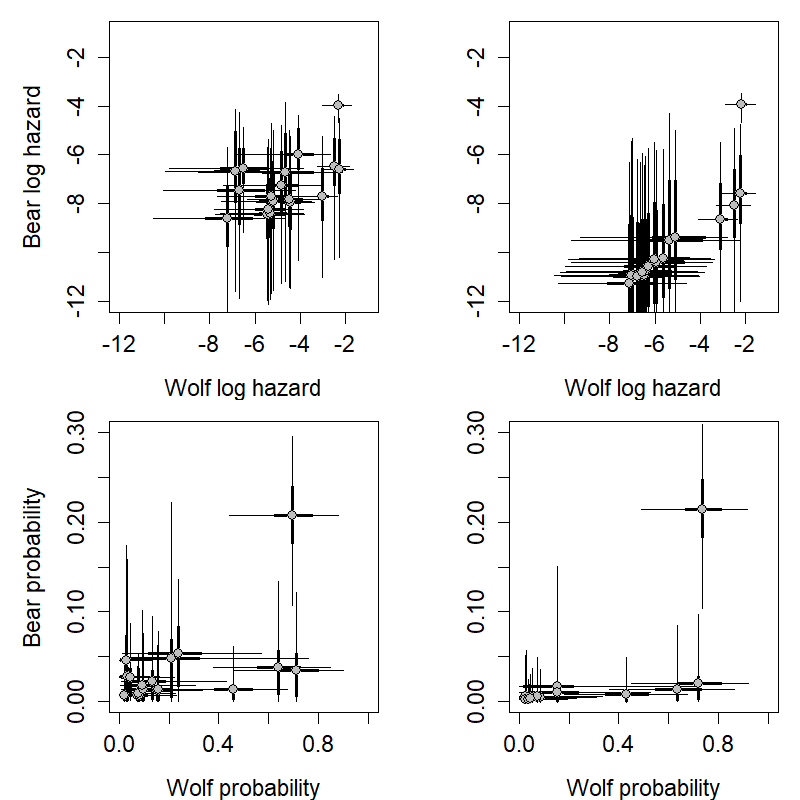


**Figure S3.** Estimated annual wolf and grizzly bear exposure hazards (log scale top row) and annual probabilities (bottom row) from a model without any diagnostic test error (Model 3, left column) and a model including diagnostic testing error (Model 5, right column). For these results we assumed a titer threshold of $\geq$16.


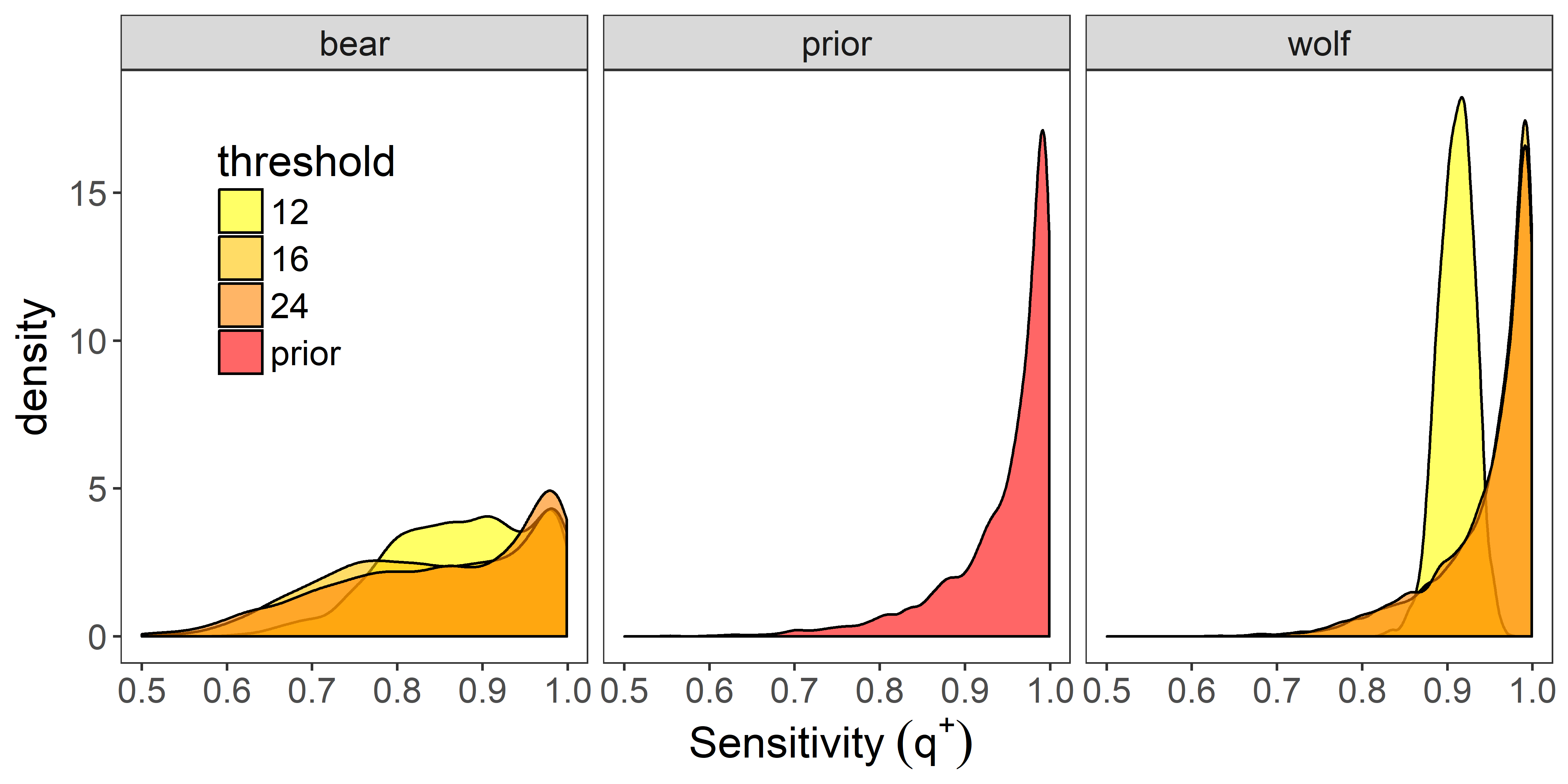

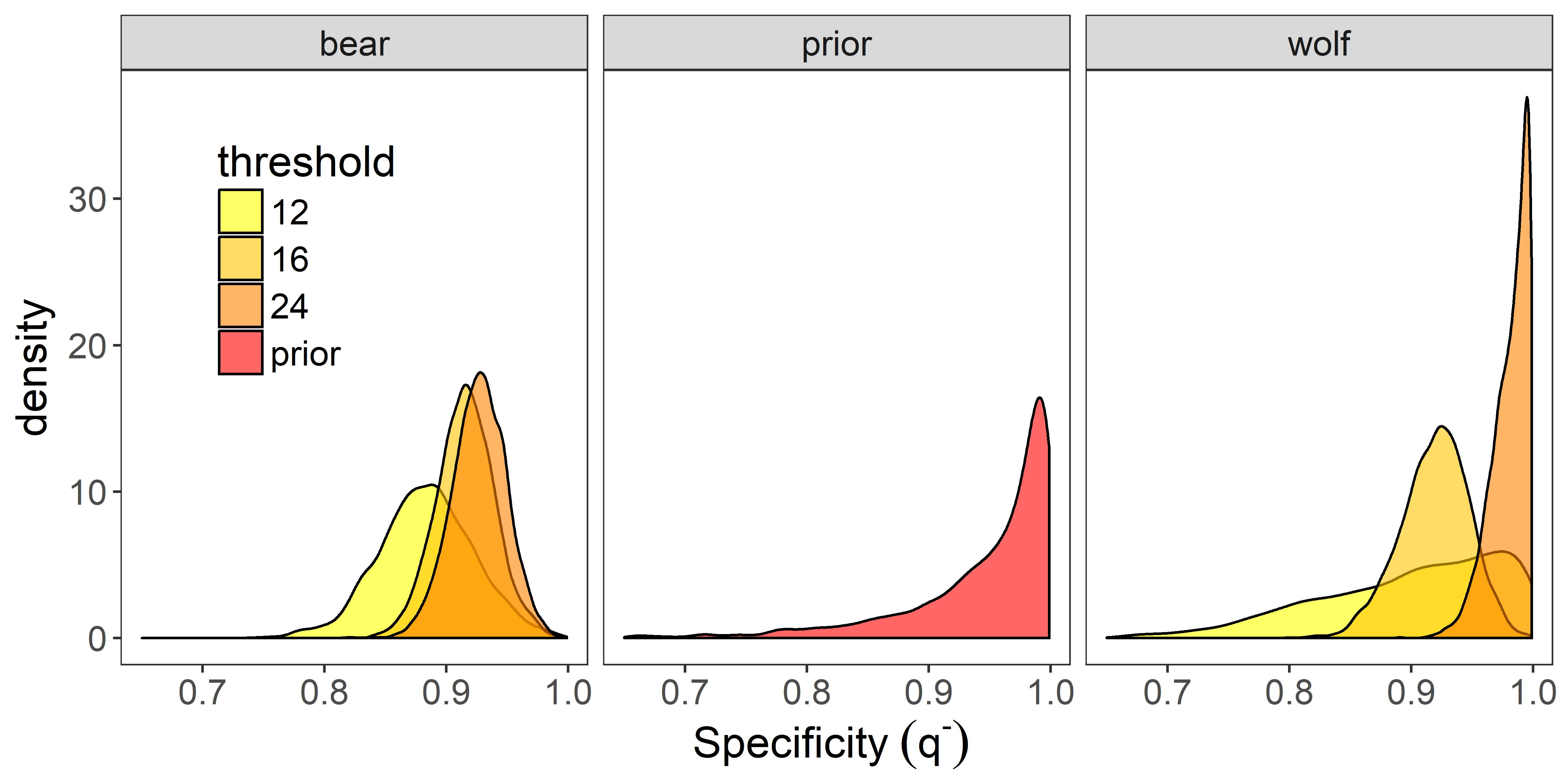


**Figure S4.** The prior and posterior distributions from Model 5.3 of the sensitivity (*q*^+^, top row) and specificity(*q*^-^, bottom row) to estimate canine distemper virus dynamics in wolves and grizzly bears in the Greater Yellowstone Ecosystem, 1984–2014. The prior distribution for both *q*^+^  and *q*^-^ was a Beta(10, 0.5).


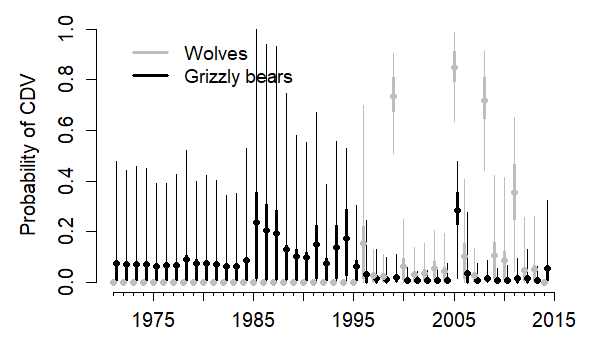


**Figure S5.** The estimated annual canine distemper virus (CDV) exposure probabilities for grizzly bears (black) and wolves (grey) assuming different serum neutralization thresholds, Greater Yellowstone Ecosystem, 1984–2014. Thick and thin lines represent the 50^th^ and 95^th^ credibility intervals, respectively. Annual estimates were based on Model 5.1 (see Table 1) using only the data from north of Yellowstone Lake assuming a titer threshold of $\geq$16.


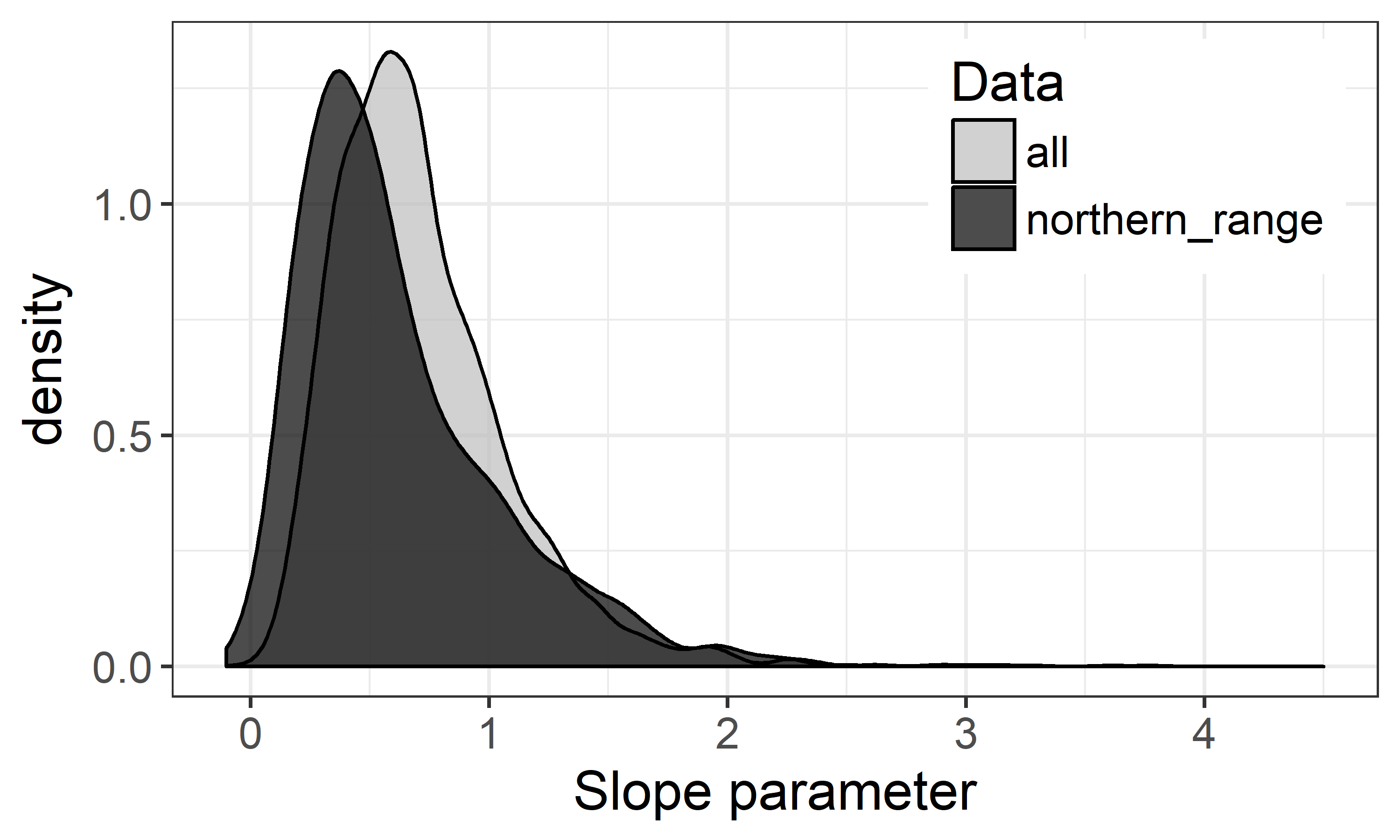


**Figure S6.** The posterior distributions of the estimated effect of wolf CDV exposure on grizzly bear exposure ($\alpha_{1}$) in the Greater Yellowstone Ecosystem did not increase when we excluded data from the southern portion of the study area (south of Yellowstone Lake) where we did not have wolf samples. Estimates based on Model 5.1 assuming a titer threshold of $\geq$16.

**Table S1**. Description of the statistical models, prior distribution and model fit assuming a serum neutralization threshold of $\geq$12 to estimate canine distemper virus dynamics in wolves and grizzly bears.**

*k* represented the year from 1 to 44, *s* = 1 for wolves and 2 for grizzly bears.

$\gamma$ is the log hazard of exposure to CDV.

pD is ‘the effective number of parameters’.

DIC is the ‘Deviance Information Criterion’, and is given by DIC = Dbar + pD = Dhat + 2 pD.

**Table S2.** Description of the statistical models, prior distribution and model fit assuming a serum neutralization threshold of $\geq$24 to estimate canine distemper virus dynamics in wolves and grizzly bears.**

*k* represented the year from 1 to 44, *s* = 1 for wolves and 2 for grizzly bears.

$\gamma$ is the log hazard of exposure to CDV.

pD is ‘the effective number of parameters’.

DIC is the ‘Deviance Information Criterion’, and is given by DIC = Dbar + pD = Dhat + 2 pD.

**Model code**

Below is code for Model 5.1 - 5.3 (Table 1) written in R version 3.3.2 (Plummer 2003, R Development Core Team 2016) to run a Just Another Gibbs Sampler (JAGS) version 4.2.0 (Plummer 2003) model of the interval censored serological data to estimate the annual probability of exposure in Grizzly bears and wolves.

Model5 <- function(){

#**********************************************

# PRIOR Distributions

#**********************************************

# slope of the effect of wolves on bears in the log hazards

# prec = is the precision of the distribution.

alpha ~ dnorm(0, prec)

# qpos and neg are the diagnostic test sensitivity and specificity,

# which may differ between bears and wolves. Beta priors are passed to # the model function.

qneg.bears ~ dbeta(beta.prior[1], beta.prior[2])

qpos.bears ~ dbeta(beta.prior[1], beta.prior[2])

qneg.wolves ~ dbeta(beta.prior[1], beta.prior[2])

qpos.wolves ~ dbeta(beta.prior[1], beta.prior[2])

# gamma.w and gamma.b are the annual log hazards for wolves and bears

# no wolf data for the first 25 years.

for(i in c(1:25, n_year)){

gamma.b[i] ~ dnorm(norm.prior[1], norm.prior[2])

gamma.w[i] <- -99.99

}

# wolf and bear hazards from 1995 onwards

for(i in 26:(n_year-1)){

gamma.w[i] ~ dnorm(norm.prior[1], norm.prior[2])

beta.b[i] ~ dnorm(norm.prior[1], norm.prior[2])

# wolf effect on the bear hazard

gamma.b[i] <- beta.b[i] + alpha * gamma.w[i]

}

# Derived parameters: translate the log hazard to annual probabilities

for(i in 1:n_year){

# monthly probabilities

Prob.mo.b[i] <- 1 - exp( -exp(gamma.b[i]))

Prob.mo.w[i] <- 1 - exp( -exp(gamma.w[i]))

# annual probabilities

Prob.yr.b[i] <- 1 - ((1 - Prob.mo.b[i])^12)

Prob.yr.w[i] <- 1 - ((1 - Prob.mo.w[i])^12)

}

#**********************************************

# Likelihood

#**********************************************

for (j in 1:n) {

for (k in left[j]:(right[j] - 1)) {

#unit cumulative hazards for each timestep

UCH[j,k] <- exp(gamma.b[lookup[k]] * bear[j] +

gamma.w[lookup[k]] * wolf[j])

}

# probability of being infected (rho) is

# 1 – minus the sum of the exp(UCHs)

rho[j] <- 1 - exp(-sum(UCH[j,left[j]:(right[j] - 1)]))

# probability of being diagnosed as exposed.

p[j]<- qpos.bears * rho[j] * bear[j] +

(1 - qneg.bears) * (1 - rho[j]) * bear[j] +

qpos.wolves * rho[j] * wolf[j] +

(1 - qneg.wolves) * (1 - rho[j]) * wolf[j]

# connecting to the observed 0/1 data

infected[j] ~ dbern(p[j])

}

}

#end model

This software has been approved for release by the U.S. Geological Survey (USGS). Although the software has been subjected to rigorous review, the USGS reserves the right to update the software as needed pursuant to further analysis and review. No warranty, expressed or implied, is made by the USGS or the U.S. Government as to the functionality of the software and related material nor shall the fact of release constitute any such warranty. Furthermore, the software is released on condition that neither the USGS nor the U.S. Government shall be held liable for any damages resulting from its authorized or unauthorized use.

**References:**

Plummer, M. 2003. JAGS: A program for analysis of Bayesian graphical models using Gibbs

sampling *in* Proceedings of the 3rd International Workshop on Distributed Statistical Computing (DSC 2003), Vienna, Austria.

R Development Core Team. 2016. R: A Language and Environment for Statistical Computing. R Foundation for Statistical Computing, Vienna, Austria.
